# Regulation of host factor γ-H2AX level and location by enterovirus A71 for viral replication

**DOI:** 10.1080/21505594.2022.2028482

**Published:** 2022-01-22

**Authors:** Jinghua Yu, Wenyan Zhang, Wenbo Huo, Xiangling Meng, Ting Zhong, Ying Su, Yumeng Liu, Jinming Liu, Zengyan Wang, Fengmei Song, Shuxia Zhang, Zhaolong Li, Xiaoyan Yu, Xiaofang Yu, Shucheng Hua

**Affiliations:** a Institute of Virology and AIDS Research, the First Hospital of Jilin University, Jilin University, Changchun, China; b Department of Experimental Pharmacology and Toxicology, School of Pharmacy, Jilin University, Changchun, China; c Medicinal Chemistry, College of Pharmacy, Changchun University of Chinese Medicine, Changchun, China; d Department of Internal Medicine, The First Hospital of Jilin University, Jilin University, Changchun, China

**Keywords:** Enterovirus A71 (EVA71), γ-H2AX level, γ-H2ax location, DNA damage response, viral genome replication, viral production

## Abstract

Numerous viruses manipulate host factors for viral production. We demonstrated that human enterovirus A71 (EVA71), a primary causative agent for hand, foot, and mouth disease (HFMD), increased the level of the DNA damage response (DDR) marker γ-H2AX. DDR is primarily mediated by the ataxia telangiectasia mutated (ATM), ATM and Rad3-related (ATR), or DNA-dependent protein kinase (DNA-PK) pathways. Upregulation of γ-H2AX by EVA71 was dependent on the ATR but not the ATM or DNA-PK pathway. As a nuclear factor, there is no previous evidence of cytoplasmic distribution of γ-H2AX. However, the present findings demonstrated that EVA71 encouraged the localization of γ-H2AX to the cytoplasm. Of note, γ-H2AX formed a complex with structural protein VP3, non-structural protein 3D, and the viral genome. Treatment with an inhibitor or CRISPR/Cas9 technology to decrease or silence the expression of γ-H2AX decreased viral genome replication in host cells; this effect was accompanied by decreased viral protein expression and virions. In animal experiments, caffeine was used to inhibit DDR; the results revealed that caffeine protected neonatal mice from death after infection with EVA71, laying the foundation for new therapeutic applications of caffeine. More importantly, in children with HFMD, γ-H2AX was upregulated in peripheral blood lymphocytes. The consistent *in vitro* and *in vivo* data on γ-H2AX from this study suggested that caffeine or other inhibitors of DDR might be novel therapeutic agents for HFMD.

## Introduction

Hand, foot, and mouth disease (HFMD) is a viral disease, which is mainly prevalent among children aged < 5 years. The cardinal symptoms of HFMD include fever and vesicles on the hands, feet, and in the mouth, which last for seven to 10 days. In some patients, severe cardiopulmonary and neurologic complications or even death can occur [1,2]. Human enterovirus A71 (EVA71) and coxsackievirus A16 (CA16) are primary causative agents of typical HFMD in connection with major outbreaks in Asia [1]. The inactivated EVA71 vaccine elicits EVA71-specific immune responses; however, vaccination does not confer cross-protection for HFMD caused by other enteroviruses [3]. In addition, there are no effective medicines for HFMD treatment because the pathogenicity of EVA71 is not entirely elucidated.

EVA71 belongs to the family *Picornaviridae* and genus *Enterovirus*. EVA71 has a positive-sense and single stranded RNA genome (approx. 7,400 bp) that is translated into one large polyprotein, and the polyprotein is cleaved into structural proteins and non-structural proteins by viral proteases [2]. Viral structural proteins (VP4, VP2, VP3, and VP1) are responsible for forming the icosahedrally symmetric capsid whereas viral non-structural proteins (2A, 2B, 2 C, and 3A, 3B, 3C, 3D) are responsible for genomic replication, protein expression, maturation, and so on. For example, in viral genomic replication, 3D protein incorporates nucleotides into the RNA strand as an RNA-dependent RNA polymerase [4]. In the course of clarifying the pathogenesis of EVA71, investigators have researched different aspects to reveal the relationship between host cells and EVA71 [5–7]. Our previous research proved that EVA71 arrests the host cell cycle at the S phase to enhance viral production [8]. However, the precise mechanism through which EVA71 regulates the host’s cell cycle to facilitate its viral replication remains to be characterized.

The DNA damage response (DDR) senses genomic damage and activates cell cycle checkpoints to facilitate DNA repair. When cells are unable to repair the damage, apoptosis will occur. As the upstream event of the arresting cell cycle and apoptosis, DDR is usually investigated when the phenomena of cell cycle arrest and/or apoptosis appears under certain conditions. The DDR signal is primarily transduced following distinct types of damage by phosphatidylinositol 3 kinase (PI3K)-like kinases ataxia telangiectasia mutated (ATM), ATM and Rad3-related kinase (ATR), or catalytic subunit of the DNA-dependent protein kinase (DNA-PK). These kinases are recruited to sites of genomic damage to phosphorylate H2AX-S139 and form γ-H2AX foci in the nucleus. Notably, γ-H2AX foci play a functional role in cell cycle arrest, damage repair, senescence, and apoptosis [9,10]. However, numerous recent studies have suggested that the DDR has additional functions [11], especially in the viral life cycle [12–21]. For example, hepatitis B virus [12], Rift Valley fever virus [13], coronavirus [14], La Crosse encephalitis virus [15], Epstein–Barr virus [16], herpes simplex virus 1 [17], human immunodeficiency virus [18], adenovirus [19], simian virus 40 (SV40) [20], and polyomavirus [21], inhibit or promote the DDR pathway to facilitate their own replication. However, past studies have mainly focused on virus-activating DDR and/or the effect of inhibiting DDR on viral production; consequently, the interaction between viruses and γ-H2AX has not been well investigated. In this study, we examined the interaction between EVA71 and host γ-H2AX.

## Materials and methods

### Ethical statement

Mouse experiments were completed in the First Hospital of Jilin University (Changchun, China) and supported by the Animal Care and Use Committee of this hospital (2018–187). Human samples were gathered from the First Hospital of Jilin University, which got the approval of the Ethics Committee of this hospital (2018–187). In the present study informed consent as written form was provided from the parents of all children.

### The source of viruses and cells

EVA71 (the Changchun077 strain) was purified and 5.0 × 10^8^ of TCID50/ml was maintained as previous described [8,22]. EVD68 (the US/KY/14-18,953 strain) was purchased from the American Type Culture Collection (No VR-1825D, Manassas, VA, USA). CA6 (the 46 strain, GenBank Accession No. KT779410.1) was obtained from the Jilin Provincial Center for Diseases Control and Prevention (Changchun, China). CA16 (the Shzh05 strain, GenBank Accession No. EU262658) was kindly provided by Professor Qi Jin (the Institute of Pathogen Biology, Beijing, China). And the amplifications of all the viruses were performed in human rhabdomyosarcoma RD cells. RD cells (No CCL-136), human cervical cancer HeLa cells (No CCL-2^TM^) and human fetal lung fibroblast MRC-5 cells (No CCL-171^TM^) were from the American Type Culture Collection. All the cell lines were cultured in DMEM (Dulbecco’s modified Eagle’s medium; Hyclone, Logan, UT, USA) together with 10% fetal bovine serum (Biological Industries, Kibbutz Beit Haemek, Israel) in an incubator with 5% carbon dioxide at 37°C. Cell culture plates or dishes were from Guangzhou Jet Bio-Filtration Co., Ltd. (Guangzhou, China).

### Determination of viral titer

The viral titer was measured in RD cells using a micro-titration assay by detecting the median tissue culture infective dose (TCID50) as previous studies [8,23]. The viral titer was calculated in accordance with the Reed–Muench method [24], assumed condition with 1 × 10^5^ TCID50/ml would lead to 0.7 × 10^5^ plaque forming units/ml [8,23].

### Western blotting analysis

Western blotting analysis was conducted as previous methods [8,22]. Cells (1 × 10^6^) were collected and washed once with PBS, and then lysed with 100 µl of lysis buffer (P0013, Beyotime Biotech, Beijing, China). The samples of lysates were then subjected to SDS-PAGE, then proteins were transferred into vinylidene fluorise transfer membranes (Pall Cor., Ann Arbor, Michigan, USA) or nitrocellulose membranes (Bio-Rad, San Diego, Califonia, USA), and then incubated with corresponding primary antibody and horseradish peroxidize-conjugated secondary antibody or alkaline phosphatase-conjugated secondary antibody.

The following antibodies were used: anti-γ-H2AX (#9718, Cell Signaling Technology, MA, USA), anti-H2AX (10,856-1-AP, Proteintech, Wuhan, China), anti-EVA71 3D (GTX630193, GeneTex, CA, USA), anti-EVA71 VP3 (GTX132341, GeneTex), anti-EVA71 VP2 (GTX132340, GeneTex), anti-EVA71 2 C (GTX132354, GeneTex), anti-EVA71 3AB (GTX132344, GeneTex), anti-EVA71 3C (GTX132357, GeneTex), anti-CA6 VP1 (GTX132346, GeneTex), anti-EV68 VP1 (GTX132313, GeneTex), anti-GAPDH (60,004-1-Ig, Proteintech), anti-β-actin (66,009-1-Ig, Proteintech), β-Tubulin (10,068-1-AP, 10,094-1-AP, Proteintech), anti-HA (51,064-2-AP, Proteintech) and anti-Histone (17,168-1-AP, Proteintech). Polyclonal anti-VP1 antibody against CA16 or EVA71 was prepared by our laboratory. AP-conjugated-anti-Mouse (115–055-003) and AP-conjugated-anti-Rabbit (111–055-003) secondary antibodies were from the Jackson Laboratory (ME, USA). HRP-conjugated-Mouse (#7076) and Rabbit (#7074) secondary antibodies were obtained from Cell Signal Technology.

### Comet assays

After lysis, the nucleoid structure of normal cells remains under electrophoresis because these possess supercoiled DNA loops. Cells with DNA damage cannot retain the nucleoid structure because they lack intact supercoiled DNA loops. DNA fragments form a comet-like shape in agarose under electrophoresis to form a comet-like shape [25]. Alkaline comet assays were conducted as the manufacturer’s instruction (Trevigen, MD, USA). RD cells were inoculated with mock or EVA71 (multiplicity of infection [MOI] = 1) for 24 h. Subsequently, the cells were collected and maintained on ice in phosphate-buffered saline (PBS). After counting cells, these cells were diluted in PBS to 1 × 10^5^/ml, then mixed the diluted cells with low melting point agarose (agarose:cells, 10:1) at 37°C. After that, 50 µl of mixture (agarose and cells) was placed on a two-well comet slide. The slide was placed into lysate at 4°C for 2 h in dark and then placed into alkaline unwinding buffer at room temperature for 20 min. Next, in alkaline electrophoresis buffer electrophoresis at 21 V was executed for 30 min. Subsequently, the two-well comet slide was gently rinsed twice with water and once with ethanol (5 min/wash). Thereafter, the two-well comet slide was kept at 37°C to dry it, and then stained by SYBR Gold for 30 min in the dark, and visualized through fluorescence microscopy.

### The distribution of cell cycle by flow cytometry

Propidium iodide is one fluorescent DNA binding dye, the level of propidium iodide fluorescence is proportional to the DNA content in one cell, therefore cells in different phases of cell cycle can be analyzed by flowcytometry [26]. Collected cells were fixed at 4°C in 1 ml of cold 70% ethanol overnight, then the cells were gently rinsed once with cold PBS, then re-dispersed in 1 ml of propidium iodide buffer (50 mg/l propidium iodide; 20 mg/l RNase; 1× PBS) at 4°C for 2 h. Subsequently, propidium iodide-stained cells were detected with FACS (BD, NY, USA). DNA histograms data was supported by the version 2.0 of ModFit LT (Verity Software House, Marine, USA) software.

### DNA fragmentation determination

In the course of apoptosis, internucleosomal DNA fragmentation forms a “DNA ladder” pattern, which we detected using DNA agarose electrophoresis [27]. According to previous study [28], both adherent and floating RD cells (1 × 10^6^) were collected. The cell pellet was lyzed in 100 μl of buffer [0.5% Triton-100; 10 mM Tris-HCl pH 7.4; 10 mM edetic acid pH 8.0] at 4°C for 30 min. Then, lysis buffer was centrifuged at 4°C with 25,000 × g for 20 min. The supernatant was collected and kept with 2 μl of 20 g/l RNase A at 37°C for 1 h, then mixed with 2 μl of 20 g/l proteinase K at 37°C for 2 h, then added with 20 μl of NaCl (5 M) and 120 μl of isopropanol, finally kept at – 20°C overnight. The next day, the supernatant were centrifuged at 25,000 × g for 15 min at 4°C. The supernatant was discarded and sedimentary DNA content was dissolved in TE buffer (10 mM Tris-HCl pH 7.4, 1 mM EDTA pH 8.0) and separated by 2% agarose gel electrophoresis for 50 min at 100 V.

### The mRNA level detected by quantitative real-time reverse transcription-PCR

RNA content from treated cells was extracted and prepared with Trizol reagent (Gibco-BRL, Rockville, MD, USA) according to previous protocols [8,22]. With oligo dT primers, total RNA was reverse-transcribed into cDNA by the high capacity cDNA RT kit (Thermo Fisher Scientific, Waltham, MA, USA) as instruction of the manufacturer. And then template cDNA and primers were mixed with SYBR GREEN PCR Master Mix (Thermo Fisher Scientific) for quantitative PCR.

The primer sequences were as follows: H2AX, F-AGCTGCTGGGCGGCGTGACG and R-CTCCTGGGAGGCCTGGGTGG; VP1, F-AGCACCCACAGGCCAGAACACAC, and R-ATCCCGCCCTACTGAAGAAACTA; GAPDH, F-GCAAATTCCATGGCACCGT and R-TCGCCCCACTTGATTTTGG. The changes relative to the levels of GAPDH were calculated using the ∆Ct method, high ∆Ct value marked low mRNA concentration.

### Treatment with DDR inhibitor

Two h after inoculation with EVA71 (MOI = 1), cells were gently rinsed once with PBS to eliminate virions remained in the medium and then treated with different doses of ATR inhibitor (VE-821, A2521; ApexBio, TX, USA), DNA-PK inhibitor (KU-57788, S2638; Selleck, TX, USA), or ATM inhibitor (KU-55933, SC202963; Santa Cruz), respectively.

### Transfection of siRNA

The siRNA targeting ATM (siATM; GGCACAAAAUGUGAAAUUCdTdT) [29], ATR (siATR; AGAAAGGAUUGUAGGCUAAUGGAdTdT) [30], DNA-PK (siDNA-PK; CCUGAAGUCUUUACAACAUAUdTdT) (Sigma website) and negative control siRNA were prepared and tested by Sangon Biotech Co., Ltd. (Shanghai, China). RD cells were cultured for 12 h to reach about 50% confluence for transfection. In accordance with the instructions provided by the manufacturer, transIntro EL Transfection Reagent (Transgen, Beijing, China) was utilized to deliver siRNA complex into cells. At 12 h after transfection, the cells were inoculated with EVA71 (MOI = 2) for another 24 h. Subsequently, the cells were lysed and prepared for Western blotting.

### Immunofluorescence staining

RD cells were inoculated with EVA71 (MOI = 2) for 24 h. 24 h later, RD cells were fixed for 15 min by 1 ml of 4% paraformaldehyde. Subsequently, RD cells were gently washed three times with PBS (5 min/wash), then at room temperature cells were blocked in blocking buffer (0.3% Triton X-100 and 5% normal serum in PBS) for 60 min. After blocking, rabbit anti-γ-H2AX antibody (Cell Signal) was added at 4°C overnight. After three rinses with PBS (5 min/wash), RD cells were treated with fluorescein isothiocyanate-goat anti-rabbit IgG (H + L) (Cell Signal, USA) at room temperature for another 2 h. Then 5 mg/l of phalloidin-tetramethylrhodamine B isothiocyanate (Sigma) was used to counterstain cytoskeleton for 20 min, finally 10 mg/l Hoechst 33,258 (Sigma) was used to counterstain nuclear DNA for another 15 min.

### Extracting the cytoplasmic and nuclear proteins

Preparing the cytoplasmic and nuclear proteins according to the instruction (P0028, Beyotime Biotech, Beijing, China). RD cells (about 1 × 10^6^ cells) were collected, and lysed in 100 µl of cytoplasmic protein extraction buffer A, then vortex to single cell, and kept on ice for 15 min, then added 10 µl of cytoplasmic protein extraction buffer B, vortex for 5 sec, then kept on ice for 1 min, then vortex for 5 sec again, then centrifuged with 14,000 g for 5 min at 4°C. The supernatant was collected as cytoplasmic contents, and the pellets were added 25 µl of nuclear protein extraction buffer. Then cytoplasmic and nuclear proteins were detected by Western blot.

### Protein immunoprecipitation

RD cells (about 2 × 10^6^ cells/dish) were grew in two 10-cm culture dish for 24 h, and then infected with mock or EVA71 with 1 of MOI for additional 24 h. After 24 h, RD cells were gently rinsed once using cold PBS buffer, and then scraped into 1 ml of cold lysis buffer for immunoprecipitation (1% NP-40; 1 mM ethylenediaminetetraacetic acid [EDTA]; 0.25% sodium deoxycholate; 150 mM NaCl; 50 mM Tris, pH 7.4) on ice. Next, the lysis buffer was collected, and part of the lysate (100 µl) was utilized as input sample, and the γ-H2AX antibody (Santa Cruz or Abcam, Cambridge, UK) was added into the remaining cell lysate (900 µl), later protein G agarose beads were added for immunoprecipitation analysis. After immunoprecipitation, for the detection of protein-protein complexes, total protein was separated and detected by Western blotting, while for RNA-protein complexes, extracted total RNA was assessed using real-time PCR.

For plasmid transfection, HeLa cells (about 5 × 10^6^ cells/dish) were grew in three 10-cm culture dishes for 24 h. 24 h later, 10 μg of VR1012-HA (control vector), 10 μg of VR1012-3D-HA, or 10 μg of VR1012-VP3-HA were transfected into HeLa cells mixed with 30 μl of transfection reagent Lipofectamine 2000 (Invitrogen, CA, USA) for 36 h. Next, the cells were dispersed into 1 ml of lysate for immunoprecipitation, and part of the lysate (100 µl) was utilized as input sample, while Anti-HA affinity matrix (11,815,016,001, Roche, Basel, Switzerland) was added to the remaining lysates to immunoprecipitate the HA, 3D-HA and VP3-HA protein. These proteins were detected using Western blotting with γ-H2AX, VP3, 3D and GAPDH antibody.

### Plasmid construction for clustered regularly interspaced short palindromic repeats (CRISPR)/CRISPR-associated protein 9 (CRISPR/Cas9) to knock out H2AX and H2AX

To knock out human *H2AX* genes, relative gRNAs were preliminarily designed through the Optimized CRISPR Design Tool, and gRNAs (Forward: CACCGCCCACTGGGAACTGGAGGC; Reverse: AAACGCCTCCAGTTCCCAGTGGGC) were screened out and utilized in this study. Double-strand oligo DNA (control) or *H2AX* gRNA was inserted into a LentiGuide-Puro (stuffer) vector at the BsmBI site. Next, 293 T cells were transfected with this vector using a packaging mix for lentiviral packaging. Then Lentivirus were collected, and inoculated HeLa cells, at 48 h post infection, 2.5 µg/ml blasticidin (Sigma) and 1.0 µg/ml puromycin (Sigma) were added to HeLa cells in drug screen to obtain positive cells. Then, cell cloning from single cell was acquired and detected for H2AX protein expression by Western blotting with H2AX antibodies. Meanwhile, H2AX DNA sequences were detected to confirm the gene knockout. The H2AX-knockout cell line was constructed by Sangon Biotech (Shanghai) Co., Ltd.

### Human H2AX plasmid construction

Synthetic H2AX cDNA of human was inserted into a VR1012 control vector using restriction enzymes Sal I and Bam H1 (Takara, Japan).

### Neonatal mouse infection model

Neonatal mice (age: 1 day) were used in the animal experiment for viral infection. All the mice were specific-pathogen-free and purchased from the Institute of Cancer Research (Experimental Animal Center, Jilin University, Jilin province, China). The mice experiments were executed in line with the related ethical regulations and the Guide for the Care and Use of Laboratory Animals of the First Hospital of Jilin University. Neonatal mice were randomly divided into five groups; each group was 8 mice. The mice of four groups were intracerebrally inoculated with EVA71 (1.5 × 10^6^ TCID50), while those of the remaining group (negative control) were intracerebrally injected with minimal essential medium (10 µl/mouse). Meanwhile, the EVA71-inoculated mice were intraperitoneally injected with 0, 2, 5, or 10 mg/kg caffeine daily for 9 days post infection. The survival rates were kept a record each day for 9 days after EVA71 infection.

### Statistical analyses

Data are presented as means ± standard deviation. And the differences between two groups were analyzed with Student’s *t*-test, and multiple groups using the post hoc test of one-way analysis of variance, two groups with repeated measures with general linear model in software of SPSS 10.0 (SPSS Inc., IL, USA). *P*-values <0.05 denoted statistically significant differences.

## Results

### γ-H2AX levels are up-regulated after infection with EVA71 and other enteroviruses

**Figure 1.**
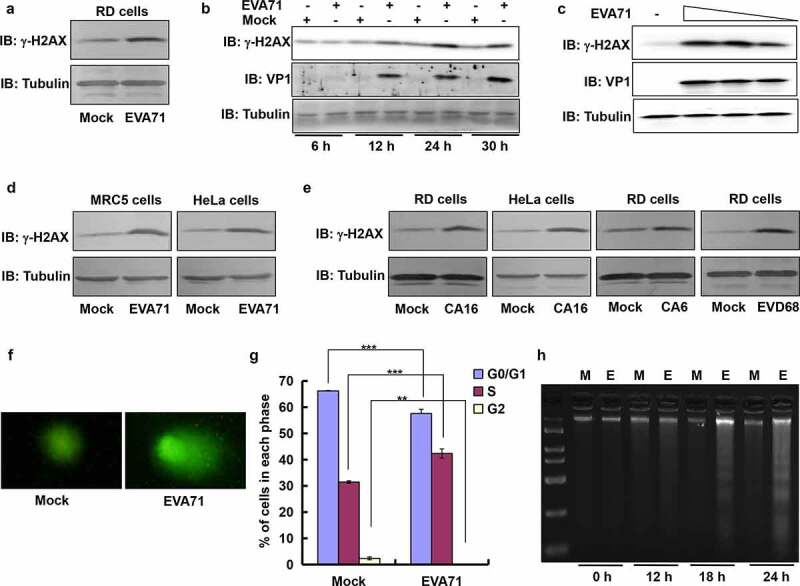
EVA71 infection up-regulated the level of γ-H2AX and induced DDR. (a) γ-H2AX expression was detected by Western blotting at 24 h post-EVA71 infection (MOI = 1) in RD cells. Tubulin is shown as loading control. (b) The expression of γ-H2AX and viral protein VP1 was detected by Western blotting at 6, 12, 24, and 30 h post-EVA71 infection (MOI = 1) in RD cells. Tubulin is shown as loading control. (c) The expression of γ-H2AX and viral protein VP1 was detected by Western blotting at 24 h post-EVA71 infection (MOI = 10, or 1, or 0.1) in RD cells. Tubulin is shown as loading control. (d) γ-H2AX expression was detected by Western blotting at 24 h post-EVA71 infection in MRC-5 cells (MOI = 2) or HeLa cells (MOI = 10). Tubulin is shown as loading control. (e) γ-H2AX expression was detected by Western blotting at 24 h post-CA16 infection in RD cells (MOI = 1) and HeLa cells (MOI = 10), or post-CA6 or post-EVD68 infection (MOI = 5, respectively) in RD cells. Tubulin is shown as loading control. (f) Alkaline comet assays were performed at 24 h post-EVA71 infection (MOI = 1). Representative cells from each sample are shown after performing three independent experiments. (g) RD cells were mock-infected or infected with EVA71 (MOI = 1). At 24 h post infection, cells were collected, and the cell-cycle profiles were analyzed using flow cytometry. The histograms were analyzed using the ModFit LT software to determine the percentage of cells in each phase of the cell cycle. ***P < 0.001. **P < 0.01. (h) RD cells mock-infected or infected with EVA71 (MOI = 1) were collected at 0, 12, 18 and 24 h post infection for analysis of DNA fragmentation through agarose gel electrophoresis. M denotes DNA marker.

CA6, coxsackievirus A6; CA16, coxsackievirus A16; DDR, DNA damage repair; EVD68, enterovirus D68; EVA71, enterovirus A71; MOI, multiplicity of infection; RD, rhabdomyosarcoma; M, Mock; E, EVA71

Viruses often manipulate the DDR in host cells [12–21,31], however it is not clear whether the DDR is induced by EVA71, which is associated with typical HFMD [32]. For prove it, we evaluated the level of phosphorylated H2AX (γ-H2AX), a well-established marker for DDR, in cells infected with EVA71. At an MOI of 1, EVA71 obviously increased the levels of γ-H2AX at 24 h post infection in human RD cells (Figure 1a). In mock-infected cells, γ-H2AX presented stable expression from 12 to 30 h post infection. However, this expression was increased significantly in RD cells infected with EVA71 between 12 and 30 h post infection (Figure 1b). Subsequently, it was confirmed that the level increase in γ-H2AX was in parallel with the titer increase of EVA71 in RD cells (Figure 1c). We sought to determine whether the upregulation of γ-H2AX was exclusive to the RD cell line. For this purpose, we selected MRC-5 (MOI = 2) and HeLa cells (MOI = 10) to further analyze the levels of γ-H2AX after infection with EVA71. The results showed that EVA71 infection upregulated the expression of γ-H2AX at 24 h post infection in MRC-5 and HeLa cells (Figure 1d). Therefore, EVA71 infection is associated with upregulation of γ-H2AX in various types of cells.

Enteroviruses are the most common human pathogens, and they share identical genomic organization and high sequence homology. To determine whether the effects of EVA71 on γ-H2AX extend to related enteroviruses, we assessed the effects of infection with CA16 (associated with typical HFMD [32]), CA6 (associated with atypical HFMD [33]), and EVD68 (associated with respiratory illness [34]). Levels of γ-H2AX in RD cells (MOI = 1) and HeLa cells (MOI = 10) were increased after infection with CA16 for 24 h, as well as after infection with CA6 (RD cells, MOI = 5) or EVD68 (RD cells, MOI = 5) for 24 h (Figure 1e). Therefore, these results suggested that the effects of enteroviruses in regulating γ-H2AX are broad. Comet electrophoresis is an important and direct method to detect DNA damage in one cell. Under electrophoresis, in cells with DNA damage DNA fragments relax to form a comet-like shape [25]. To further confirm the induction of the DDR in RD cells, comet electrophoresis was performed at 24 h post EVA71 infection (MOI = 1). The observed comet tail demonstrated that infection with EVA71 induced the DDR (figure 1f). A common consequence of DDR induction includes the activation of cell cycle checkpoints together with repair of damaged DNA or induction of apoptosis [35]. Therefore, we analyzed the cell cycle with flow cytometry after propidium iodide staining and apoptosis with DNA agarose electrophoresis by observing DNA ladder after EVA71 infection [8,22]. In cell cycle analysis, it was found that infection with EVA71 in RD cells induced cell cycle arrest in S phase (*P* < 0.001), companied with reduced G0/G1 (*P* < 0.001) and G2/M (*P* < 0.01) (Figure 1g) at 24 h post infection. In apoptotic analysis, an obvious DNA ladder appeared in DNA agarose electrophoresis with infection time whereas intact DNA was present in mock-infected RD cells (Figure 1h). Therefore, EVA71 induced cell cycle arrest and apoptosis; these results were consistent with those of our previous studies [8,22]. Thus, EVA71 induces the DDR and upregulates γ-H2AX.

### EVA71 upregulates γ-H2AX via the ATR pathway

**Figure 2.**
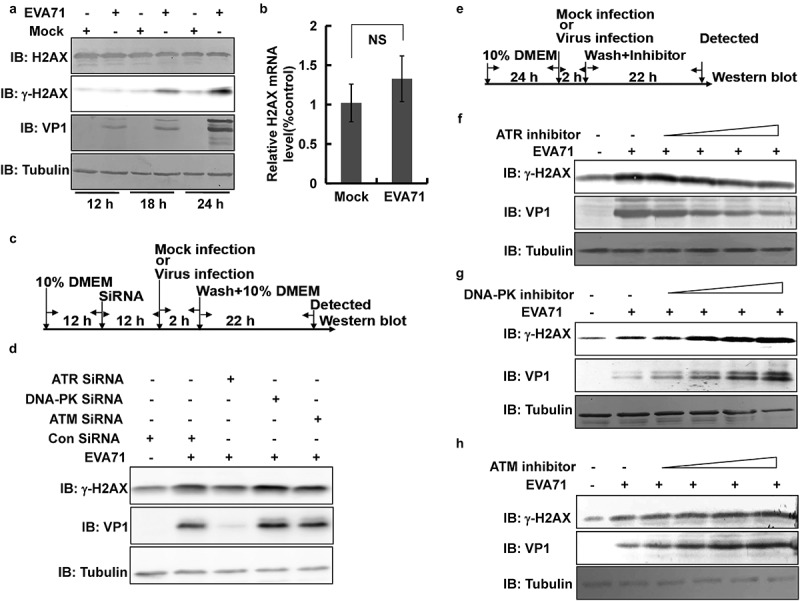
ATR pathway was responsible for H2AX phosphorylation and γ-H2AX formation.. (a) RD cells mock-infected or infected with EVA71 (MOI = 1) were collected at 12, 18, and 24 h post infection to detect the expression of H2AX, γ-H2AX, and VP1 proteins through Western blotting. Tubulin is shown as loading control. (b) At 24 h post-EVA71 infection (MOI = 1), the mRNA levels of H2AX were detected using real-time quantitative PCR. The results were standardized to the levels of GAPDH and normalized to 1 in mock-infected cells. Data are represented as the mean ± standard deviation of three independent experiments. NS: no significant difference. (c) The flow diagram showing the method through which RD cells were transfected with control siRNA, siATR, siDNA-PK, or siATM; 12 h later, cells were infected with EVA71 (MOI = 2) for 2 h; at 24 h post infection, the cells were collected for Western blotting analysis. (d) The expression of γ-H2AX and VP1 proteins was assessed by Western blotting after (c) treatment. Tubulin is shown as loading control. (e) Flow diagram showing the method through which RD cells were treated with inhibitors, namely ATR inhibitor (VE-821), DNA-PK inhibitor (KU 57788), or ATM inhibitor (KU 55933) after EVA71 infection (MOI = 1) at 2 h post infection. (F–H) At 2 h post-EVA71 infection, ATR inhibitor (VE-821; 0, 2, 4, 8, or 16 µM) was added for an additional 22 h (f); DNA-PK inhibitor (KU 57788; 0, 1.3, 2.5, 5, or 10 µM) was added for an additional 18 h (g); and ATM inhibitor (KU 55933; 0, 1.3, 2.5, 5, or 10 µM) was added for an additional 18 h (h); subsequently, the cells were collected for Western blotting analysis. Tubulin is shown as loading control. ATM, ataxia telangiectasia mutated; ATR, ATM and Rad3-related; EVA71, enterovirus 71; GAPDH, glyceraldehyde-3-phosphate dehydrogenase; MOI, multiplicity of infection; PCR, polymerase chain reaction; RD, rhabdomyosarcoma; siATM, siRNA targeting ATM; siATR, siRNA targeting ATR; siDNA-PK, siRNA targeting DNA-PK

γ-H2AX is formed following the phosphorylation of serine at position 139 of H2AX [14]. Thus, we examined whether EVA71 upregulated γ-H2AX by increasing the levels of γ-H2AX precursor. The analysis showed that upregulation of γ-H2AX was not accompanied by a change in the expression of H2AX in RD cells (Figure 2a). Moreover, the mRNA levels of H2AX were not affected by EVA71 infection versus mock infection at 24 h post infection in RD cells (Figure 2b). Therefore, the upregulation of γ-H2AX by EVA71 may be mediated by phosphorylation. H2AX is often phosphorylated by the ATR, DNA-PK, or ATM pathways to form γ-H2AX [14]. To confirm the role of ATR, DNA-PK, or ATM in the regulation of γ-H2AX expression, RD cells were transfected with siATR, siDNA-PK, or siATM. At 12 h post transfection, the cells were infected with EVA71 at an MOI of 2 for 2 h. At 24 h post infection, the cells were collected for Western blotting analysis (Figure 2c and S1a). Unlike siDNA-PK and siATM, siATR inhibited the upregulation of γ-H2AX and VP1 upon infection with EVA71 (Figure 2d and S1b).

To further evaluate the effect of these pathways on γ-H2AX expression, we treated RD cells with an ATR inhibitor (VE-821), DNA-PK inhibitor (KU 57788), or ATM inhibitor (KU 55933) at 2 h post infection with EVA71 (MOI = 2). At 24 h post infection, the cells were collected for Western blotting analysis (Figure 2e). At increasing doses (0, 2, 4, 8, and 16 µM), the ATR inhibitor inhibited the upregulation of γ-H2AX induced by infection with EVA71; importantly, the ATR inhibitor also inhibited the expression of viral protein VP1 (figure 2f). However, treatment with the DNA-PK inhibitor (0, 1.3, 2.5, 5, and 10 µM) (Figure 2g) or the ATM inhibitor (0, 1.3, 2.5, 5, and 10 µM) (Figure 2h) did not decrease the levels of γ-H2AX in RD cells infected with EVA71; on the contrary, γ-H2AX expression was increased in a dose-dependent manner by each of these (Figures 2g and 2h). Furthermore, the expression of VP1 was increased in parallel with the dose of the DNA-PK inhibitor (Figure 2g and S2) or ATM inhibitor (Figure 2h and S3). To further confirm the activation and role of ATR in viral production, the ATR inhibitor schisandrin B (5 µM, N1656; ApexBio) was also used. The results demonstrated that schisandrin B also decreased levels of γ-H2AX and inhibited viral production (Figure S4) to a similar extent as VE-821 (8 µM; Figure S5). Therefore, the ATR pathway is responsible for the phosphorylation of H2AX, and there is a positive correlation with γ-H2AX level and viral production. Additionally, the ATM and DNA-PK pathways might be inactivated and there is a negative correlation with γ-H2AX level and viral production.

### γ-H2AX was localized to the cytoplasm after infection with EVA71

**Figure 3.**
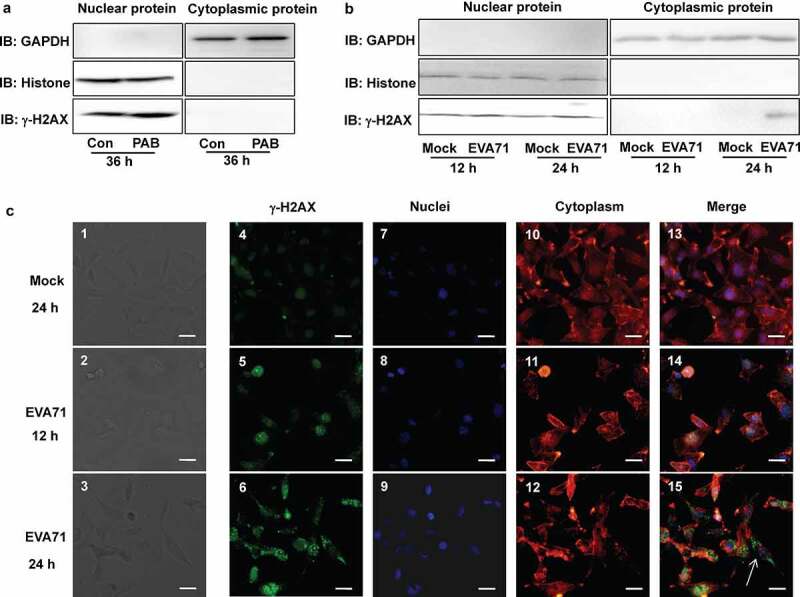
γ-H2AX was localized to the nucleus and cytoplasm after EVA71 infection. (a) The nuclear or cytoplasmic protein of RD cells was extracted, and the level of γ-H2AX was detected by Western blotting after treatment with the positive control pseudolaric acid B (4 µM) for 36 h. Histone and GAPDH are shown as loading controls for nuclear and cytoplasmic proteins, respectively. (b) The nuclear or cytoplasmic protein of RD cells was extracted, and the level of γ-H2AX was detected by Western blotting after EVA71 infection (MOI = 1) for 12 and 24 h. Histone and GAPDH are shown as loading controls for nuclear and cytoplasmic proteins, respectively. (c) γ-H2AX distribution analysis by fluorescence microscopy at 24 h post infection. Cells were stained with γ-H2AX antibodies (Cell Signal), and co-stained with FITC labeled-secondary antibody (green). DNA was counterstained with Hoechst 33,258 (blue). Cytoskeleton was counterstained with phalloidin-tetramethylrhodamine B isothiocyanate (Red). Merged images of γ-H2AX with DNA are shown. And the corresponding white light image was kept. The arrow indicated cell with normal morphology and irregular cytoplasmic location. Scale bar: 15 µm. EVA71, enterovirus A71; FITC, fluorescein isothiocyanate; GAPDH, glyceraldehyde-3-phosphate dehydrogenase; MOI, multiplicity of infection

Previous studies have demonstrated the nuclear distribution of γ-H2AX [9]. Hence, in this study, we investigated the location of γ-H2AX in the nucleus after infection with EVA71. We selected pseudolaric acid B (a diterpene acid) as a positive control because it induces the DDR [36]; subsequently, we extracted nuclear and cytoplasmic proteins for Western blotting analysis. The results showed that levels of γ-H2AX protein were increased in the nucleus at 36 h after treatment with 4 µM pseudolaric acid in RD cells (Figure 3a). Surprisingly, however, at 24 h post infection with EVA71, the γ-H2AX protein exhibited a cytoplasmic distribution in RD cells (Figure 3b).

Given that this was the first report of the cytoplasmic distribution of γ-H2AX, we sought to confirm our results by performing immunofluorescence analysis. In this analysis, we verified that at 24 h post infection, mock-infected RD cells had intact morphology (Figure 3c1), and at 12 h post infection, EVA71-infected cells still had intact morphology (Figure 3c2); at 24 h, EVA71-infected cells became smaller (Figure 3c3). Infection with EVA71 increased the levels of γ-H2AX protein in cells over time (Figures 3c6 and 3c5 vs. 3c4). The nuclei became condensed and brighter after EVA71 infection (Figures 3c9 and 3c8 vs. 3c7) whereas the cytoskeleton appeared to collapse with infection time (Figures 3c12 and 3c11 vs. 3c10). Importantly, after 12-h infection, γ-H2AX was still distributed in the nuclei of EVA71-infected cells (Figures 3c14 and 3c13), whereas at 24 h post infection, γ-H2AX was mainly distributed in the cytoplasm (Figure 3c15). Therefore, after EVA71 infection, γ-H2AX was first upregulated in the nuclei, after which γ-H2AX was located in the cytoplasm. Furthermore, nuclei condensed cells were more evident with high levels of γ-H2AX (Figures 3c6 and 3c9) and with high rates of viral replication in RD cells (Figure S6a). These results are the first to indicate that γ-H2AX is located in the cytoplasm after infection with EVA71.

### γ-H2AX co-localized with viral genome and viral proteins VP3 and 3D after infection with EVA71

**Figure 4.**
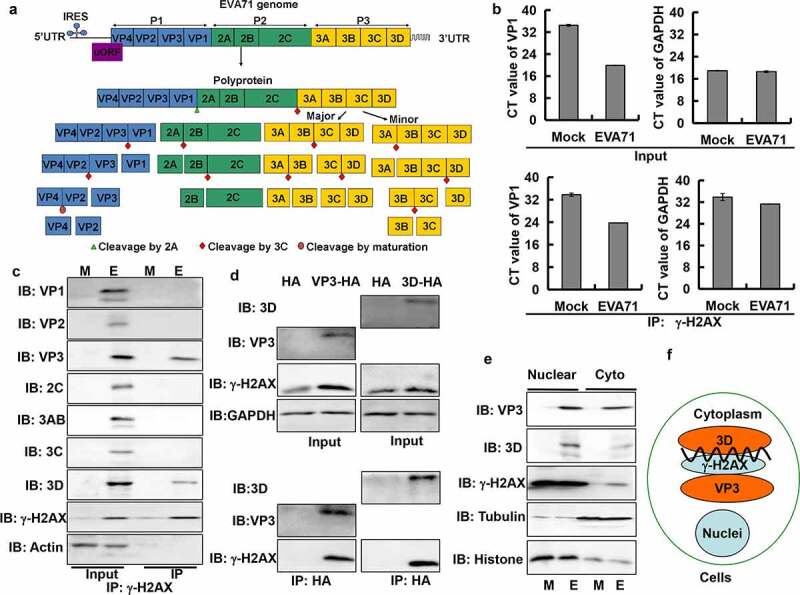
γ-H2AX co-localized with viral genome, viral protein VP3, and 3D. (a) The scheme of viral genome and viral protein. The viral RNA genome encodes one large polyprotein which is cleaved into precursors and finally four structural proteins, and seven non-structural proteins by 2A protease, 3 C protease or maturation. (b) EVA71 genomic content in RD cells was evaluated by RT-PCR analysis of VP1 mRNA before and after immunoprecipitation (IP) with γ-H2AX antibody (Santa). CT values of VP1 and GAPDH in input sample and IP sample with γ-H2AX antibody are shown. Data are represented as the mean ± standard deviation of three independent experiments. (c) The levels of viral proteins (VP1, VP2, VP3, 2 C, 3AB, 3 C, and 3D) in RD cells infected with EVA71 were evaluated by Western blotting before and after immunoprecipitation (IP) with γ-H2AX antibody (Cell Signal). Actin is shown as loading control. (d) HeLa cells were collected at 36 h after transfection with 10 μg of VR1012-VP3-HA (VP3-HA), VR1012-3D-HA (3D-HA) or the corresponding control vector VR1012-HA (HA). Anti-HA affinity matrix was used for immunoprecipitation of HA-tagged proteins, which were analyzed by Western blotting with γ-H2AX, VP3 and 3D antibody. The results are representative of three independent experiments. (e) The nuclear and cytoplasmic proteins of RD cells were extracted, and the levels of γ-H2AX, VP3, and 3D were detected using Western blotting after EVA71 infection (MOI = 1) for 24 h. Histone and tubulin are shown as loading controls for nuclear and cytoplasmic proteins, respectively. (f) Diagram showing that γ-H2AX forms a complex with the viral genome, viral protein VP3, and 3D in cells infected with EVA71. CT, cycle threshold; EVA71, enterovirus A71; HA, hemagglutinin; MOI, multiplicity of infection; RD, rhabdomyosarcoma; RT-PCR, reverse-transcription polymerase chain reaction; M, mock; E, EVA71

EVA71 is a positive single strand RNA virus. The viral RNA genome is replicated in cytoplasmic replication centers, and is translated into one large polyprotein, which is cleaved into precursors and finally four structural proteins (VP1, VP2, VP3 and VP4), and eight non-structural proteins (UP, 2A, 2B, 2 C, 3A, 3B, 3 C and 3D) by 2A protease, 3 C protease, or maturation [2] (Figure 4a). Given reports that human papillomavirus accumulates γ-H2AX in viral replication centers [37] and the fact that γ-H2AX was localized in the cytoplasm where EVA71 replication occurs, we hypothesized that γ-H2AX might interact with the cytoplasmic viral genome. To test this hypothesis, we collected RD cellular content for real-time PCR and evaluated the CT value as a measure of the limit of detection; CT values >30 indicate that the mRNA sample is negative [38,39]. In the input sample, the CT value of VP1 in cells infected with EVA71 was approximately 19.9 ± 0.05 whereas in mock-infected cells, it was 34.5 ± 0.34 (which is >30). Therefore, the specificity of virus collection was confirmed. Meanwhile, in the input sample, the CT values of GAPDH for mock-infected and EVA71-infected cells were the same; hence, equivalent levels of cellular content were verified prior to immunoprecipitation (Figure 4b). Furthermore, immunoprecipitates of γ-H2AX protein had a substantially lower VP1 CT value in EVA71-infected RD cells (23.73 ± 0.06) than in mock-infected cells (33.79 ± 0.58), indicating that the EVA71 genome was bound to γ-H2AX in the cells. The CT values of GAPDH in EVA71-infected cells and mock-infected cells were both >30, ruling out nonspecific binding (Figure 4b). Therefore, it was shown that γ-H2AX bound to the genome of EVA71 in cells.

Subsequently, we hypothesized that γ-H2AX might interact with viral protein(s) to participate in EVA71 production. Immunoprecipitation of γ-H2AX affirmed that the protein bound to viral structural protein VP3 and non-structural 3D protein in EVA71-infected RD cells; however, it did not bind with VP1, VP2, 2 C, 3AB, or 3 C protein. In addition, there was no expression of β-actin in the immunoprecipitation samples (Figure 4c). The absence of antibodies for VP4, 2A, 2B, and UP made their detection impossible. The results showed that γ-H2AX protein particularly bound with VP3 and 3D proteins. To further confirm the binding of viral protein with γ-H2AX, VP3-HA or 3D-HA was correctly overexpressed (Figure S6b) in HeLa cells, which are easily transfected with plasmids. Immunoprecipitates of VP3-HA or 3D-HA protein were collected with Anti-HA Affinity Matrix (Roche), and the expression of γ-H2AX was assessed by Western blotting analysis. Overexpression of VP3 or 3D increased the expression of γ-H2AX at 36 h post transfection (Figure 4d). Further immunoprecipitation analysis confirmed that γ-H2AX bound with VP3 and 3D (Figure 4d), which was consistent with the results shown in Figure 4c. Moreover, VP3, 3D, and γ-H2AX were distributed synchronously in the nucleus and cytoplasm of RD cells (Figure 4e). Although it remains unclear whether this binding is direct, the present findings indicate that γ-H2AX, viral genome, VP3 protein, and 3D protein form a complex in the cytoplasm (Figure 4f).

### Knockout of H2AX inhibited viral genome replication and viral production

**Figure 5.**
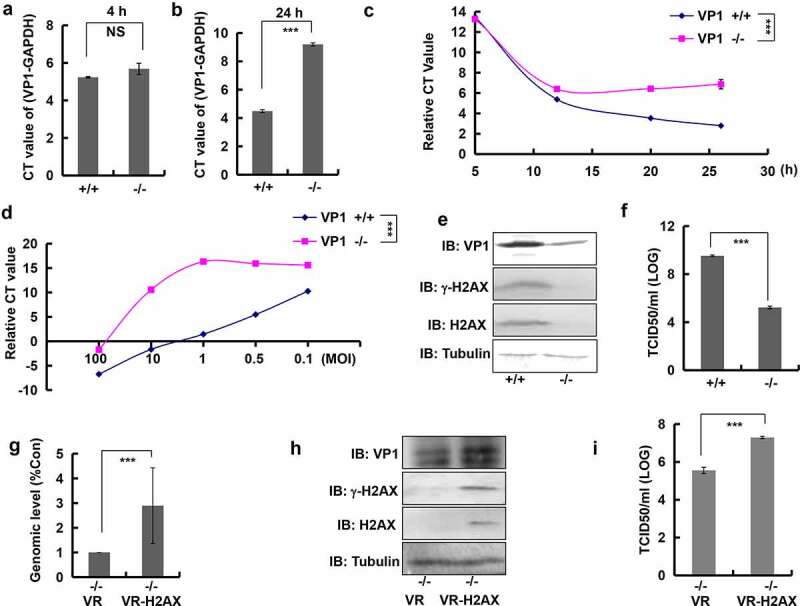
Knockout of H2AX decreased EVA71 genome replication and viral production. (A, B) At 4 h (a) or 24 h (b) post-EVA71 infection (MOI = 10), the mRNA levels of VP1 were detected using real-time quantitative PCR. The results were standardized to the levels of GAPDH. Data are represented as the mean ± standard deviation (SD) of three independent experiments. ***P < 0.001. NS, no significant difference. (c) At 5, 12, 20, and 26 h post-EVA71 infection (MOI = 10), the mRNA levels of VP1 or GAPDH were detected using real-time quantitative PCR. And the relative CT values (VP1-GAPDH) were shown. Data are represented as the mean ± SD of three independent experiments. ***P < 0.001. (d) At 36 h post-EVA71 infection (MOI = 100, 10, 1, 0.5 and 0.1), the mRNA levels of VP1 and GAPDH were detected using real-time quantitative PCR. The relative CT values (VP1-GAPDH) are also shown. Data are represented as the mean ± SD of three independent experiments. ***P < 0.001. (e) The expression of VP1, γ-H2AX, H2AX, and tubulin in wild-type and knockout cells at 24 h post-EVA71 infection (MOI = 10). (F) The TCID50 was assessed at 24 h post-EVA71 infection (MOI = 10). Log10^TCID50/mL^ is shown. Data are presented as the mean ± SD of three independent experiments. ***P < 0.001. (G–I) Knockout cells were transfected with VR1012 plasmid or VR1012-H2AX plasmid. At 2 h post transfection, the cells were inoculated with EVA71 (MOI = 10) for 24 h. (g) The mRNA levels of VP1 and GAPDH were detected using real-time quantitative PCR. The results were standardized to the levels of GAPDH and normalized to 1 in cells transfected with VR1012. Data are represented as the mean ± SD of three independent experiments. ***P < 0.001. (h) Western blotting analysis of VP1, γ-H2AX, and H2AX expression. Tubulin is shown as loading control. (i) The TCID50 was assessed at 24 h post infection. Data are presented as the mean ± SD of three independent experiments. ***P < 0.001. +/+, wild type; −/−, knockout H2AX; CT, cycle threshold; EVA71, enterovirus 71; GAPDH, glyceraldehyde-3-phosphate dehydrogenase; MOI, multiplicity of infection; PCR, polymerase chain reaction; TCID50, median tissue culture infective dose

To highlight the effect of γ-H2AX on viral production, we created stable H2AX-knockout cells. This was achieved using CRISPR/Cas9 technology to delete the T base at position 71 in HeLa cells (Figure S6c). These cells lost their ability to express H2AX or γ-H2AX protein (Figure S6d). Next, we investigated viral genome replication; the amount of viral genome was equivalent in wild-type and knockout cells at 4 h after infection with EVA71 (MOI = 10), indicating that γ-H2AX did not inhibit viral entry (Figure 5a). However, at 24 h, the relative CT of (VP1-GAPDH) was changed from 4.49 ± 0.11 to 9.20 ± 0.10 in knockout cells versus wild-type cells (Figure 5b). Therefore, these results confirm that γ-H2AX is related to viral replication.

We also observed that in wild-type cells, the viral genome was continually replicating from 5 h to 26 h; the relative CT values (VP1-GAPDH) were 13.46 ± 0.10, 5.38 ± 0.03, 3.53 ± 0.02, 2.79 ± 0.03 at 5, 12, 20, and 26 h post infection, respectively. In knockout cells, the viral genome was replicated only in the first 12 h, and the viral genome was unchanged from 12 h to 26 h after infection; the relative CT values were 13.27 ± 0.05, 6.40 ± 0.03, 6.42 ± 0.01, and 6.87 ± 0.46 at 5, 12, 20, and 26 h post infection, respectively (Figure 5c), indicating blockage of viral genome replication in the absence of γ-H2AX.

To further support the role of γ-H2AX in viral replication, serial dilutions of viruses were used. At 36 h post infection, the relative CT values in wild-type cells treated with decreasing MOIs (100, 10, 1, 0.5, and 0.1) were −6.72 ± 0.05, −1.64 ± 0.07, 1.5 ± 0.02, 5.55 ± 0.02, and 10.16 ± 0.02, respectively, and the relative CT values increased with decreasing MOIs, indicating that the viral genome level was positively correlated to the MOI, and virus with either a high or low MOI could replicate. In knockout cells, the relative CT values were −1.73 ± 0.16, 10.56 ± 0.07, 16.33 ± 0.29, 15.94 ± 0.39, and 15.60 ± 0.73 with decreasing MOIs, respectively (Figure 5d), indicating that the viral genome level was not positively correlated to the MOI for viruses with a low MOI (1, 0.5, and 0.1); viruses with a low MOI could not replicate as well as those with a high MOI. These findings indicated that viral replication requires γ-H2AX, highlighting its importance in this process. Subsequently, the expression of viral proteins was inhibited in knockout cells (Figure 5e). At 24 h post infection with EVA71 (MOI = 10), the TCID50 was decreased from 3.38 ± 0.53 × 10^9^ to 1.72 ± 0.43 × 10^5^ in knockout cells versus wild-type cells (figure 5f). Therefore, a decrease in γ-H2AX expression also inhibited viral production.

To further confirm the role of γ-H2AX in viral production, knockout cells were transfected with the VR1012 or VR012-H2AX plasmid and infected with EVA71 (MOI = 10) for 24 h. The results showed that regaining of H2AX increased viral genome replication (*P* < 0.001) (Figure 5g), VP1 expression (Figure 5h), and viral production (*P* < 0.001) (Figure 5i). More importantly, the expression of H2AX was accompanied by the formation of γ-H2AX (Figure 5h). Therefore, γ-H2AX plays an important role in viral genome replication and viral production.

### Caffeine decreased the levels of γ-H2AX and protected neonatal mice from damage induced by EVA71

**Figure 6.**
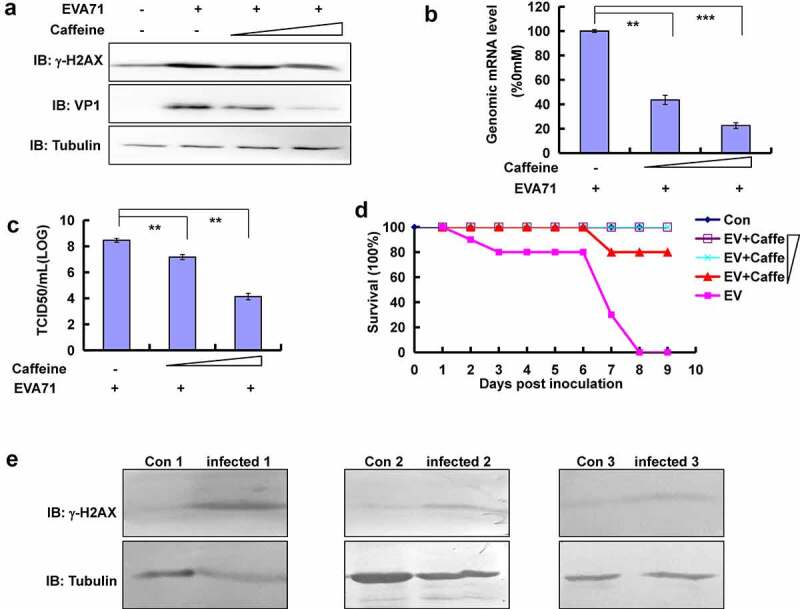
Confirmation of the relationship between γ-H2AX and EVA71 viral production *in vitro and in vivo*. (a) Caffeine (1 or 2 mM) was added to cells 2 h after infection with EVA71, and cell lysates were collected 24 h later for Western blotting analysis of VP1 and γ-H2AX expression. Tubulin is shown as loading control. (b) Pre-treatment with different doses of caffeine (0, 1, and 2 mM) for 12 h. Cells were washed once with phosphate-buffered saline (PBS) and infected with EVA71 (MOI = 1) for 2 h; subsequently, the cells were re-treated with different doses of caffeine (0, 1, and 2 mM) for 10 h. Genomic mRNA levels were detected using real-time quantitative PCR by targeting the VP1 sequence. The results were standardized to the levels of GAPDH and normalized to 100 in infected cells treated with 0 mM caffeine. Data are presented as the mean ± SD of three independent experiments. **P < 0.01, and ***P < 0.001. (c) The TCID50 was assessed at 28 h post-EVA71 infection (MOI = 1). Log10^TCID50/mL^ is shown. Data are represented as the mean ± SD of three independent experiments. (d) Protective effect of caffeine against lethal challenge with EVA71 *in vivo*. Mice of the four groups were intracerebrally inoculated with 1.5 × 10^6^ TCID50 of EVA71, while those of the remaining group were intracerebrally inoculated with DMEM (10 µl/mouse). At the same time, EVA71-inoculated mice were intraperitoneally injected with 0, 2, 5, or 10 mg/kg caffeine daily for 9 days post infection, and the survival rates were monitored daily. Kaplan–Meier plots were used to calculate survival. Con: mock-infected and treated with 0 mg/kg caffeine; EV: EVA71-infected and treated with 0 mg/kg caffeine; EV+Caffe: EVA71-infected and treated with 2 mg/kg, 5 mg/kg, or 10 mg/kg caffeine. (e) The expression of γ-H2AX in children with HFMD. Blood samples were collected from control and infected children, and the levels of γ-H2AX in peripheral blood mononuclear cells were determined using Western blotting. Tubulin is shown as loading control. The results are representative of three independent experiments. Con, healthy children aged <6 years; DMEM, Dulbecco’s modified Eagle’s medium; EVA71, enterovirus 71; GAPDH, glyceraldehyde-3-phosphate dehydrogenase; HFMD, hand, foot, and mouth disease; Infected, children with infection; MOI, multiplicity of infection; PCR, polymerase chain reaction; SD, standard deviation; TCID50, median tissue culture infective dose

Caffeine is widely used in *in vitro* [14,40] and *in vivo* [41,42] studies of the DDR because it can inhibit the formation of γ-H2AX [14,40]. To further determine the role of γ-H2AX in the production of EVA71, caffeine at 1 mM and 2 mM doses was used in subsequent analysis. Of note, at these doses, caffeine did not affect cell proliferation (data not shown). Western blotting assays demonstrated that an increase in the dose of caffeine (1 or 2 mM) led to an obvious decrease in the levels of γ-H2AX and viral protein expression at 24 h post infection in a dose-dependent manner in RD cells (Figure 6a). Furthermore, reverse transcription-PCR of cellular lysates suggested that the inhibition occurs in RD cells at viral replication (<5-fold for 1 mM caffeine and <10-fold for 2 mM caffeine) (Figure 6b). As assessed by the TCID50, caffeine decreased viral production in RD cells in a dose-dependent manner (18.86-fold and 19,735-fold reduction caused with 1 mM and 2 mM caffeine, respectively) (Figure 6c). Therefore, caffeine decreased the levels of γ-H2AX and reduced viral genome replication and viral production *in vitro*.

More importantly, caffeine protected neonatal mice from death induced by infection with EVA71 in a dose-dependent manner (Figure 6d) *in vivo*. Owing to its limited toxicity, caffeine has been used in clinical practice for the treatment of headaches [43]. Moreover, caffeine can inhibit viral multiplication and protect host cells. Based on these effects, caffeine may be useful as an anti-HFMD drug.

### γ-H2AX was upregulated in children with HFMD

To confirm the clinical importance of γ-H2AX, we collected blood samples from patients with HFMD (Figure S7) and measured the levels of γ-H2AX in peripheral blood mononuclear cells extracted using lymphocyte separation liquid (Corning, NY, USA). Nevertheless, children with HFMD exhibited increased levels of γ-H2AX compared with the control group (healthy children aged <6 years) (Figure 6e), which was consistent with our cell culture results. Therefore, γ-H2AX is clinically significant.

## Discussion

EVA71, one of the primary causative pathogens of HFMD, is associated with recent outbreaks in Asia [1,2]. Our previous results confirmed that infection with EVA71 induced cell cycle arrest at the S phase [8] and apoptosis [22], and that these are usually caused by the DDR pathway, which is a signaling network that senses and repairs cellular DNA lesions [35]. Thus, we investigated whether EVA71 induced DDR, and proved that infection with EVA71 upregulated γ-H2AX and induced DDR. Moreover, other enteroviruses such as CA16, CA6, and EVD68, also had the ability to activate the DDR pathway.

As previously reported, DNA viruses and retroviruses such as the herpes simplex virus [17], human papillomavirus [44], human immunodeficiency virus [18], and polyomavirus [21] usually initiate the DDR pathway. However, prior to the present study, only a limited number of RNA viruses such as hepatitis C virus [45], coronavirus [14], La Crosse encephalitis virus [15], Rift Valley fever virus [13], Newcastle disease virus [46], and rotavirus [47] had been confirmed to activate the DDR pathway and upregulate the expression of γ-H2AX. In viral life cycle, DNA viruses and retroviruses must integrate their DNA by inserting it into the host genome, which leads to a direct DNA break. However, most RNA viruses replicate in the cytoplasm but still induce DDR. Therefore, it is speculated that the viral protein transported into the nucleus might cause indirect or direct DNA damage; thus, viral protein(s) of EVA71 involved in DDR pathways need further investigation.

In eukaryotes, histones H2A, H2B, H3, and H4 form nucleosomes with approximately 150 bp DNA. With DNA damage, the serine at position 139 of H2AX, an H2A variant, is phosphorylated and H2AX becomes p-H2AX-S139 (γ-H2AX) [9,10]. In this study, infection with EVA71 upregulated the expression of γ-H2AX; however, this did not affect the expression of H2AX, indicating that EVA71 infection might promote H2AX phosphorylation at position 139. H2AX phosphorylation pathways are activated by distinct types of damage [9], and ATM, ATR, and DNA-PK are the three main kinases involved in these pathways. DNA-PK and ATM are primarily involved in DNA double-strand breaks whereas ATR primarily responds to DNA single-strand breaks [9]. It was confirmed that siATR blocked the upregulation of γ-H2AX after EVA71 infection whereas siATM or siDNA-PK promoted the upregulation of γ-H2AX after EVA71 infection. We found that the ATR inhibitor decreased γ-H2AX levels in a dose-dependent manner whereas the ATM or DNA-PK inhibitor increased γ-H2AX levels in a dose-dependent manner. Therefore, the ATR pathway differs from the ATM and DNA-PK pathways with respect to EVA71 replication. EVA71 might induce DNA single-strand breaks to activate the ATR pathway which is responsible for the formation of γ-H2AX after EVA71 infection whereas the activation of ATM or DNA-PK might be lowered after EVA71 infection. Furthermore, inhibition of the ATR pathway decreased γ-H2AX levels and inhibited virus production whereas inhibition of the ATM and DNA-PK pathways increased γ-H2AX levels and promoted virus production. Therefore, virus production is positively correlated with γ-H2AX levels and ATR activation. However, γ-H2AX levels are negatively correlated with ATM and DNA-PK activation after EVA71 infection. Viral proteins or the viral genome might form a special spatial structure to regulate ATM, ATR, or DNA-PK and lead to DDR.

The expression of γ-H2AX, but not that of H2AX, is closely related to virus production. Moreover, EVA71 infection regulates γ-H2AX levels but not H2AX levels; therefore, the interaction between γ-H2AX and virus production is being investigated. To date, γ-H2AX has been perceived as a nuclear protein for the repair of damaged DNA; however, two detection methods (immunofluorescence staining and Western blotting of nuclear and cytoplasmic fractions) have shown that γ-H2AX is localized to the cytoplasm after EVA71 infection. This evidence is the first in its field to support the localization of γ-H2AX protein in the cytoplasm after viral infection.

As EVA71 replication occurs in the cytoplasm, we hypothesized that γ-H2AX protein may be co-located in the cytoplasm with viral proteins or the viral genome. It was demonstrated that γ-H2AX protein bound to the viral structural protein VP3, and non-structural protein 3D, as well as to the EVA71 genome. Although we could not confirm whether the binding was direct, γ-H2AX formed complexes with the viral genome and viral VP3 and 3D proteins in the cytoplasm. This might explain the induction of DDR pathways caused by EVA71 infection, the localization of γ-H2AX in the cytoplasm, and the role of γ-H2AX in virus production.

To highlight the effect of γ-H2AX on virus production, we generated H2AX-knockout cells. We observed that although EVA71 could normally enter these knockout cells, its genome replication was substantially inhibited. In knockout cells, the viral genome was replicated only in the first 12 h after viral infection. By contrast, in wild-type cells, the viral genome was continuously amplified for 26 h. Serial dilutions of a virus stock were used to further investigate the role of γ-H2AX in viral replication. At 36 h post infection, all diluted viruses could replicate in wild-type cells treated with decreasing MOI (100, 10, 1, 0.5, 0.1). However, in knockout cells, only viruses with an MOI of 100 and 10 could replicate effectively. These findings indicate that the absence of γ-H2AX affects viral replication; and thus, γ-H2AX is important for viral genome replication.

Further investigation also revealed that viral protein expression and TCID50 were also decreased in H2AX-knockout cells. More importantly, re-expression of the H2AX protein in knockout cells through transfection with an H2AX plasmid was accompanied by the formation of γ-H2AX, as well as an increase in viral genome replication, viral protein expression, and virus production. Therefore, γ-H2AX is essential for viral genome replication and virus production.

To assess the role of γ-H2AX in EVA71, we inhibited its expression using caffeine; this approach has been extensively applied for the *in vitr*o [14,40] and *in vivo* [41,48] studies of DDR pathways. We confirmed that caffeine inhibited viral genome replication, viral protein expression, and viral production *in vitro*. More importantly, we observed the protective effect of caffeine on EVA71-infected neonatal mice *in vivo*. Considering that caffeine is used clinically to treat headache because of its low toxicity [43], caffeine may be useful as an anti-HFMD drug.

To further support the relationship between γ-H2AX and HFMD, we collected blood samples from children with and without HFMD. In patients with HFMD, we found that γ-H2AX was upregulated, thus supporting our *in vitro* findings.

Typically, γ-H2AX is used as a biomarker of the DDR pathway as it plays an important role in repairing DDR. Moreover, the results of this study confirmed that γ-H2AX inhibition could directly inhibit viral genome replication and virus production. Hence, in addition to its involvement in the DDR pathway, γ-H2AX also has an important role in viral replication, particularly because H2AX deletion does not prevent the expression of other proteins related to DDR, including upstream protein(s) of γ-H2AX. Therefore, these results show that EVA71 directly and primarily uses γ-H2AX for its viral replication and production, which differs from the findings of previous studies focusing on the effect of DDR on virus production.

Therefore, we conclude that γ-H2AX forms a complex with the EVA71 genome and viral VP3 and 3D proteins, and regulates viral genome replication and production of the viral progeny. These effects are important for the prevention and treatment of HFMD in the clinical setting.

## Supplementary Material

Supplemental Material

## Data Availability

The datasets used and/or analyzed during the current study are available from the corresponding author on reasonable request. The data used to support the findings of this study are available at 10.6084/m9.figshare.17091017 10.6084/m9.figshare.16640005 10.6084/m9.figshare.17091026 10.6084/m9.figshare.17471078 10.6084/m9.figshare.17471450 10.6084/m9.figshare.17091032 10.6084/m9.figshare.16640020 10.6084/m9.figshare.16640026

## References

[1] Chan KP, Goh KT, Chong CY, et al. Epidemic hand, foot and mouth disease caused by human enterovirus 71, Singapore. Emerg Infect Dis. 2003;9(1):78–85. 12533285 10.3201/eid1301.020112 PMC2873753

[2] Solomon T, Lewthwaite P, Perera D, et al. Virology, epidemiology, pathogenesis, and control of enterovirus 71. Lancet Infect Dis. 2010;10(11):778–790. 20961813 10.1016/S1473-3099(10)70194-8

[3] Hu Y, Zeng G, Chu K, et al. Five-year immunity persistence following immunization with inactivated enterovirus 71 type (EV71) vaccine in healthy children: a further observation. Hum Vaccin Immunother. 2018;14(6):1517–1523. 29482422 10.1080/21645515.2018.1442997 PMC6037439

[4] Baltimore D. In vitro synthesis of viral rna by the poliovirus rna polymerase. Proc Natl Acad Sci U S A. 1964;51(3):450–456. 14171458 10.1073/pnas.51.3.450 PMC300093

[5] Lei X, Xiao X, Xue Q, et al. Cleavage of interferon regulatory factor 7 by enterovirus 71 3C suppresses cellular responses. J Virol. 2012;87(3):1690–1698. 23175366 10.1128/JVI.01855-12 PMC3554134

[6] Yang CH, Li HC, Jiang JG, et al. Enterovirus type 71 2A protease functions as a transcriptional activator in yeast. J Biomed Sci. 2010;17(1):65. 20682079 10.1186/1423-0127-17-65 PMC2923119

[7] Liu Y, Wu J, Liu W. Enterovirus 71 induces neural cell apoptosis and autophagy through promoting ACOX1 downregulation and ROS generation. Virulence. 2020;11:537–553. 32434419 10.1080/21505594.2020.1766790 PMC7250321

[8] Yu J, Zhang L, Ren P, et al. Enterovirus 71 mediates cell cycle arrest in S phase through non-structural protein 3D. Cell Cycle. 2015;14(3):425–436. 25659038 10.4161/15384101.2014.980631 PMC4353240

[9] Polo SE, Jackson SP. Dynamics of DNA damage response proteins at DNA breaks: a focus on protein modifications. Genes Dev. 2011;25(5):409–433. 21363960 10.1101/gad.2021311 PMC3049283

[10] Weitzman MD, Fradet-Turcotte A. Virus DNA replication and the host DNA damage response. Annu Rev Virol. 2018;5(1):141–164. 29996066 10.1146/annurev-virology-092917-043534 PMC6462412

[11] Turinetto V, Giachino C. Multiple facets of histone variant H2AX: a DNA double-strand-break marker with several biological functions. Nucleic Acids Res. 2015;43(5):2489–2498. 25712102 10.1093/nar/gkv061 PMC4357700

[12] Luo J, Luckenbaugh L, Hu H, et al. Involvement of host ATR-CHK1 pathway in hepatitis B virus covalently closed circular DNA formation. mBio. 2020;11(1):e03423–19. 32071277 10.1128/mBio.03423-19 PMC7029148

[13] Baer A, Austin D, Narayanan A, et al. Induction of DNA damage signaling upon Rift Valley fever virus infection results in cell cycle arrest and increased viral replication. J Biol Chem. 2012;287(10):7399–7410. 22223653 10.1074/jbc.M111.296608 PMC3293538

[14] Xu LH, Huang M, Fang SG, et al. Coronavirus infection induces DNA replication stress partly through interaction of its nonstructural protein 13 with the p125 subunit of DNA polymerase delta. J Biol Chem. 2011;286(45):39546–39559. 21918226 10.1074/jbc.M111.242206 PMC3234778

[15] Verbruggen P, Ruf M, Blakqori G, et al. Interferon antagonist NSs of La Crosse virus triggers a DNA damage response-like degradation of transcribing RNA polymerase II. J Biol Chem. 2011;286(5):3681–3692. 21118815 10.1074/jbc.M110.154799 PMC3030371

[16] Nikitin PA, Yan CM, Forte E, et al. An ATM/Chk2-mediated DNA damage-responsive signaling pathway suppresses Epstein-Barr virus transformation of primary human B cells. Cell Host Microbe. 2010;8(6):510–522. 21147465 10.1016/j.chom.2010.11.004 PMC3049316

[17] Edwards TG, Bloom DC, Fisher C. The ATM and Rad3-related (ATR) Protein Kinase Pathway is Activated by Herpes Simplex Virus 1 (HSV-1) and Required for Efficient Viral Replication. J Virol. 2018;92(6):e01884–17. 29263259 10.1128/JVI.01884-17 PMC5827400

[18] Ariumi Y, Turelli P, Masutani M, et al. DNA damage sensors ATM, ATR, DNA-PKcs, and PARP-1 are dispensable for human immunodeficiency virus type 1 integration. J Virol. 2005;79(5):2973–2978. 15709017 10.1128/JVI.79.5.2973-2978.2005 PMC548471

[19] Brestovitsky A, Nebenzahl-Sharon K, Kechker P, et al. The Adenovirus E4orf4 Protein Provides a Novel Mechanism for Inhibition of the DNA Damage Response. PLoS Pathog. 2016;12(2):e1005420. 26867009 10.1371/journal.ppat.1005420 PMC4750969

[20] Zhao X, Madden-Fuentes RJ, Lou BX, et al. Ataxia telangiectasia-mutated damage-signaling kinase- and proteasome-dependent destruction of Mre11-Rad50-Nbs1 subunits in Simian virus 40-infected primate cells. J Virol. 2008;82(11):5316–5328. 18353955 10.1128/JVI.02677-07 PMC2395194

[21] Jiang M, Zhao L, Gamez M, et al. Roles of ATM and ATR-mediated DNA damage responses during lytic BK polyomavirus infection. PLoS Pathog. 2012;8(8):e1002898. 22952448 10.1371/journal.ppat.1002898 PMC3431332

[22] Song F, Yu X, Zhong T, et al. Caspase-3 Inhibition Attenuates the Cytopathic Effects of EV71 Infection. Front Microbiol. 2018;9:817. 29755438 10.3389/fmicb.2018.00817 PMC5932146

[23] Gay RT, Belisle S, Beck MA, et al. An aged host promotes the evolution of avirulent coxsackievirus into a virulent strain. Proc Natl Acad Sci U S A. 2006;103(37):13825–13830. 16950876 10.1073/pnas.0605507103 PMC1564236

[24] Reed LJML. A simple method of estimating fifty percent endpoints. Am J Hyg. 1983;27:493–497.

[25] Olive PL, Banáth JP. The comet assay: a method to measure DNA damage in individual cells. Nat Protoc. 2006;1(1):23–29. 17406208 10.1038/nprot.2006.5

[26] Crowley LC, Chojnowski G, Waterhouse NJ. Measuring the DNA Content of Cells in Apoptosis and at Different Cell-Cycle Stages by Propidium Iodide Staining and Flow Cytometry. Cold Spring Harb Protoc. 2016;2016(10). 10.1101/pdb.prot087247 27698234

[27] Majtnerová P, Roušar T. An overview of apoptosis assays detecting DNA fragmentation. Molecular Biology Reports. 2018;45(5):1469–1478. 30022463 10.1007/s11033-018-4258-9

[28] Li Z, Yu J, Liu L, et al. Coxsackievirus A16 infection induces neural cell and non-neural cell apoptosis in vitro. PLoS One. 2014;9(10):e111174. 25350381 10.1371/journal.pone.0111174 PMC4211689

[29] Hennig J, McShane MP, Cordes N, et al. APPL proteins modulate DNA repair and radiation survival of pancreatic carcinoma cells by regulating ATM. Cell Death Dis. 2014;5(4):e1199. 24763056 10.1038/cddis.2014.167 PMC4001316

[30] Luo Y, Chen AY, Qiu J. Bocavirus infection induces a DNA damage response that facilitates viral DNA replication and mediates cell death. J Virol. 2011;85(1):133–145. 21047968 10.1128/JVI.01534-10 PMC3014208

[31] Deng X, Xu P, Zou W, et al. DNA Damage Signaling Is Required for Replication of Human Bocavirus 1 DNA in Dividing HEK293 Cells. J Virol. 2016;91(1):e01831–16. 27733644 10.1128/JVI.01831-16 PMC5165215

[32] Fan X, Jiang J, Liu Y, et al. Detection of human enterovirus 71 and Coxsackievirus A16 in an outbreak of hand, foot, and mouth disease in Henan Province, China in 2009. Virus Genes. 2012;46(1):1–9. 23080402 10.1007/s11262-012-0814-x

[33] Gao F, Mao QY, Chen P, et al. Seroepidemiology of Coxsackievirus A6, Coxsackievirus A16, and Enterovirus 71 infections in infants and children: a prospective cohort study in Jiangsu, China. J Infect. 2016;73(5):509–512. 27546063 10.1016/j.jinf.2016.08.008

[34] Wang ZY, Zhong T, Wang Y, et al. Human Enterovirus 68 Interferes with the Host Cell Cycle to Facilitate Viral Production. Front Cell Infect Microbiol. 2017;7:29. 28229049 10.3389/fcimb.2017.00029 PMC5296350

[35] Gentile M, Latonen L, Laiho M. Cell cycle arrest and apoptosis provoked by UV radiation-induced DNA damage are transcriptionally highly divergent responses. Nucleic Acids Res. 2003;31(16):4779–4790. 12907719 10.1093/nar/gkg675 PMC169943

[36] Song F, Yu X, Zhang H, et al. Pseudolaric acid B inhibits neuroglioma cell proliferation through DNA damage response. Oncol Rep. 2017;38(4):2211–2218. 28765951 10.3892/or.2017.5861

[37] Reinson T, Toots M, Kadaja M, et al. Engagement of the ATR-dependent DNA damage response at the human papillomavirus 18 replication centers during the initial amplification. J Virol. 2013;87:951–964. 23135710 10.1128/JVI.01943-12 PMC3554080

[38] Zhou J, Otter JA, Price JR, et al. Investigating SARS-CoV-2 surface and air contamination in an acute healthcare setting during the peak of the COVID-19 pandemic in London. Clin Infect Dis. 2021;73(7):e1870–e1877 . 32634826 10.1093/cid/ciaa905 PMC7454437

[39] Garg A, Ghoshal U, Patel SS, et al. Evaluation of seven commercial RT-PCR kits for COVID-19 testing in pooled clinical specimens. J Med Virol. 2021;93(4):2281–2286. 33230819 10.1002/jmv.26691 PMC7753435

[40] Mitchell JK, Friesen PD. Baculoviruses modulate a proapoptotic DNA damage response to promote virus multiplication. J Virol. 2012;86(24):13542–13553. 23035220 10.1128/JVI.02246-12 PMC3503141

[41] Damiani AP, Garcez ML. A reduction in DNA damage in neural tissue and peripheral blood of old mice treated with caffeine. Journal of Toxicology and Environmental Health. Part A. 2017;80(13–15):621–629. 28524728 10.1080/15287394.2017.1286901

[42] Mercer JR, Gray K, Figg N, et al. The Methyl Xanthine Caffeine Inhibits DNA Damage Signaling and Reactive Species and Reduces Atherosclerosis in ApoE−/−Mice. Arterioscler Thromb Vasc Biol. 2012;32(10):2461–2467. 22859494 10.1161/ATVBAHA.112.251322

[43] Lipton RB, Diener HC, Robbins MS, et al. Caffeine in the management of patients with headache. J Headache Pain. 2017;18(1):107. 29067618 10.1186/s10194-017-0806-2 PMC5655397

[44] Fradet-Turcotte A, Bergeron-Labrecque F, Moody CA, et al. Nuclear accumulation of the papillomavirus E1 helicase blocks S-phase progression and triggers an ATM-dependent DNA damage response. J Virol. 2011;85(17):8996–9012. 21734051 10.1128/JVI.00542-11 PMC3165840

[45] Machida K, McNamara G, Cheng KT, et al. Hepatitis C virus inhibits DNA damage repair through reactive oxygen and nitrogen species and by interfering with the ATM-NBS1/Mre11/Rad50 DNA repair pathway in monocytes and hepatocytes. J Immunol. 2010;185(11):6985–6998. 20974981 10.4049/jimmunol.1000618 PMC3101474

[46] Ren S, Ur Rehman Z, Gao B. ATM-mediated DNA double-strand break response facilitated oncolytic Newcastle disease virus replication and promoted syncytium formation in tumor cells. PLoS Pathogens. 2020;16(6):e1008514. 32479542 10.1371/journal.ppat.1008514 PMC7263568

[47] Sarkar R, Patra U, Lo M, et al. Rotavirus activates a noncanonical ATM-Chk2 branch of DNA damage response during infection to positively regulate viroplasm dynamics. Cellular Microbiology. 2020;22(3):e13149. 31845505 10.1111/cmi.13149

[48] Li R, Zhu J, Xie Z, et al. Conserved herpesvirus kinases target the DNA damage response pathway and TIP60 histone acetyltransferase to promote virus replication. Cell Host Microbe. 2011;10:390–400. 22018239 10.1016/j.chom.2011.08.013 PMC3253558

