# Supplementary figures and images for: Regulation of host factor γ-H2AX level and location by enterovirus A71 for viral replication
Source: Virulence. 2022 Jan 22;13(1):241–57. doi: 10.1080/21505594.2022.2028482 (PMC8786350; doi:10.1080/21505594.2022.2028482)

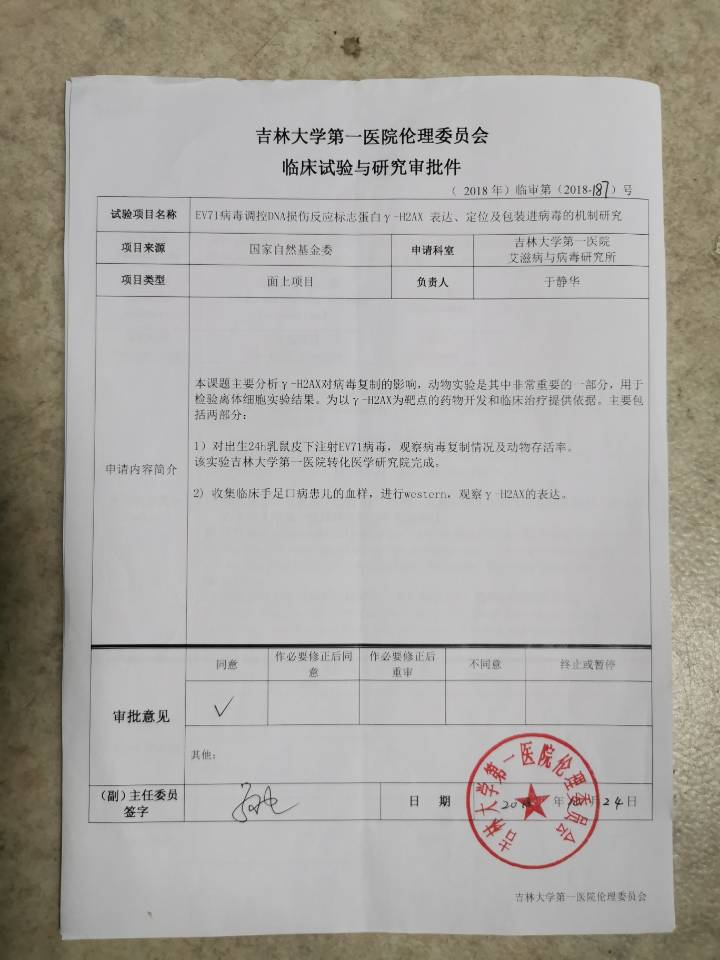

Supplement: Supplemental Material [file KVIR_A_2028482_SM2193.zip › supplementary/Ethics.jpg]

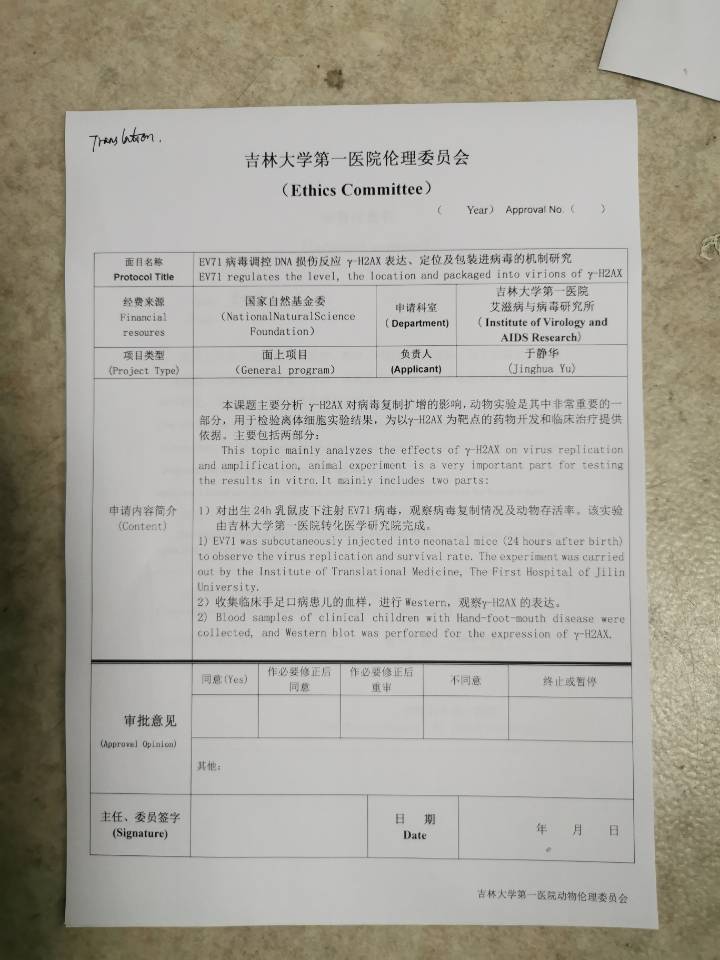

Supplement: Supplemental Material [file KVIR_A_2028482_SM2193.zip › supplementary/Ethics_translation.jpg]
