# Supplementary material for: Regulation of host factor γ-H2AX level and location by enterovirus A71 for viral replication
Source: Virulence. 2022 Jan 22;13(1):241–57. doi: 10.1080/21505594.2022.2028482 (PMC8786350; doi:10.1080/21505594.2022.2028482)
Supplement: Supplemental Material [file KVIR_A_2028482_SM2193.zip › supplementary/S-1.doc]

**Supporting information**

**S1 Fig. ATM SiRNA, ATR SiRNA and DNA-PK SiRNA decreased the level of ATM, ATR and DNA-PK, respectively.**

(A) ATM SiRNA, ATR SiRNA and DNA-PK SiRNA decreased the level of ATM, ATR and DNA-PK, respectively. (B) The gray values of Figure 2D.*P < 0.05.***P < 0.01.

**S2 Fig. DNA-PK inhibitor (KU 57788) inhibited viral production in RD cells.** At 2 h post-infection of EV71 (MOI of 1), 0 µM or 5 µM of DNA-PK inhibitor (KU 57788) was added for an additional 18 h. (A) At 20 h post infection, morphologic analysis of the effect of DNA-PK inhibitor on cell death after EV71 infection. Cell morphology was visualized by light microscopy. Arrows indicate dead cells. Bar=60 μm. The results are representative of three independent experiments. (B) At 20 h post infection, RD cell survival was evaluated by MTT analysis, and the results indicated the mean ± S.D. of three independent experiments. ***P < 0.001. (C) VP1 and γ-H2AX expression in cellular lysates was determined after growth in control medium or DNA-PK inhibitor medium at 20 h post-infection. Tubulin is loading control. The results are representative of three independent experiments. (D) At 20 h post infection, intracellular EV71 RNA levels were detected in control or 5 µM of DNA-PK inhibitor-treated RD cells by real-time quantitative PCR. The results are standardized to GAPDH mRNA as a control and normalized to 1.0 in control cells. ***P < 0.001. (E) Total progeny viruses (supernatant and cellular viruses) were titrated using RD cells at 20 h post-infection. Log TCID50 was shown. The results indicate the means ± S.D. of three independent experiments. **P < 0.01. Mock: mock infection, Con: control treatment, EV: EV71 infection, DNA-PK In: DNA-PK inhibitor.

**S3 Fig. ATM inhibitor (KU 55933) inhibited viral production in RD cells.** At 2 h post-infection of EV71 (MOI of 1), 0 µM or 5 µM of ATM inhibitor (KU 55933) was added for an additional 18 h. (A) At 20 h post infection, morphologic analysis of the effect of ATM inhibitor on cell death after EV71 infection. Cell morphology was visualized by light microscopy. Arrows indicate dead cells. Bar=60 μm. The results are representative of three independent experiments. (B) At 20 h post infection, RD cell survival was evaluated by MTT analysis, and the results indicated the mean ± S.D. of three independent experiments. **P < 0.01. (C) VP1 and γ-H2AX expression in cellular lysates was determined after growth in control medium or ATM inhibitor medium at 20 h post-infection. Tubulin is loading control. The results are representative of three independent experiments. (D) At 20 h post infection, intracellular EV71 RNA levels were detected in control or 5 µM of ATM inhibitor-treated RD cells by real-time quantitative PCR. The results are standardized to GAPDH mRNA as a control and normalized to 1.0 in control cells. ***P < 0.001. (E) Total progeny viruses (supernatant and cellular viruses) were titrated using RD cells at 20 h post-infection. Log TCID50 was shown.The results indicate the means ± S.D. of three independent experiments. **P < 0.01. Mock: mock infection, Con: control treatment, EV: EV71 infection, ATM In: ATM inhibitor.

**S4 Fig. ATR inhibitor (Schisandrin B) inhibited viral production in RD cells.** At 2 h post-infection of EV71 (MOI=1), 0 µM or 5 µM of ATR inhibitor (Schisandrin B) was added for an additional 22 h. (A) At 24 h post infection, morphologic analysis of the effect of ATR inhibitor on cell death after EV71 infection. Cell morphology was visualized by light microscopy. Arrows indicate dead cells. Bar=60 μm. The results are representative of three independent experiments. (B) At 24 h post infection, RD cell survival was evaluated by MTT analysis, and the results indicated the mean ± S.D. of three independent experiments. *P < 0.05. (C) VP1 and γ-H2AX expression in cellular lysates was determined after growth in control medium or ATR inhibitor medium at 24 h post-infection. Tubulin is loading control. The results are representative of three independent experiments. (D) At 24 h post infection, intracellular EV71 RNA levels were detected in control or 5 µM of ATR inhibitor-treated RD cells by real-time quantitative PCR. The results are standardized to GAPDH mRNA as a control and normalized to 1.0 in control cells. ***P < 0.001. (E) Total progeny viruses (supernatant and cellular viruses) were titrated using RD cells at 24 h post-infection. Log TCID50 was shown. The results indicate the means ± S.D. of three independent experiments. *P < 0.05. Mock: mock infection, Con: control treatment, EV: EV71 infection, ATR In: ATR inhibitor.

**S5 Fig. ATR inhibitor (VE-821) inhibited viral production in RD cells.** At 2 h post-infection of EV71 (MOI of 1), 0 µM or 8 µM of ATR inhibitor (VE-821) was added for an additional 22 h. (A) At 24 h post infection, morphologic analysis of the effect of ATR inhibitor on cell death after EV71 infection. Cell morphology was visualized by light microscopy. Arrows indicate dead cells. Bar=60 μm. The results are representative of three independent experiments. (B) At 24 h post infection, RD cell survival was evaluated by MTT analysis, and the results indicated the mean ± S.D. of three independent experiments. ***P < 0.001. (C) VP1 and γ-H2AX expression in cellular lysates was determined after growth in control medium or ATR inhibitor medium at 24 h post-infection. Tubulin is loading control. The results are representative of three independent experiments. (D) At 24 h post infection, intracellular EV71 RNA levels were detected in control or 8 µM of ATR inhibitor-treated RD cells by real-time quantitative PCR. The results are standardized to GAPDH mRNA as a control and normalized to 1.0 in control cells. ***P < 0.001. (E) Total progeny viruses (supernatant and cellular viruses) were titrated using RD cells at 24 h post-infection. Log TCID50 was shown. The results indicate the means ± S.D. of three independent experiments. *P < 0.05. Mock: mock infection, Con: control treatment, EV: EV71 infection, ATR In: ATR inhibitor.

**S6 Fig. The VP1 distribution and the T base of 71 were detected.** (A) EV71 VP1 distribution analysis by fluorescence microscopy at 24 h post-infection. Cells were stained with VP1 antibodies (Genetex), and then co-stained FITC labeled-secondary antibody (Green). DNA was counterstained with Hoechst 33258 (Blue). Merged images of VP1 with DNA are shown. Scale bar = 15 µm. (B)VR1012-3D-HA and VR1012-VP3-HA express the correct protein. At 24 h post infection with dose of VR1012-3D-HA and VR1012-VP3-HA, 293T cells were collected for western blot for 3D and VP3 expression with 3D, VP3 together with HA antibody. (C) The T base of 71 was deleted by CRISPR/Cas9 technology after DNA sequence analysis in different batches of knock out H2AX HeLa cells. (D) In knockout cells, there is no the expression of H2AX by western blot analysis.
