# Supplementary material for: Regulation of host factor γ-H2AX level and location by enterovirus A71 for viral replication
Source: Virulence. 2022 Jan 22;13(1):241–57. doi: 10.1080/21505594.2022.2028482 (PMC8786350; doi:10.1080/21505594.2022.2028482)
Supplement: Supplemental Material [file KVIR_A_2028482_SM2193.zip › supplementary/S-2.doc]

**S7 Table 1 Clinical manifestations of EV71 infected patients.**

| **No.of sample** | **Infected child 1** | **Infected child 2** | **Infected child 3** |
| --- | --- | --- | --- |
| **Sex** | Female | Female | Male |
| **Age** | 1Y | 3Y | 2Y |
| **Clinical manifestation** | Fever | Fever | Fever |
|  | Skin vesicular | Skin vesicular | Skin vesicular |
|  | Oral ulcer | Oral ulcer | Oral ulcer |
|  | Myoclonic jerk | Vomiting | Vomiting |
| **Complication** | No | Pulmonary edema | No |
| **Diagnosis** | HFMD | HFMD | HFMD |
| **Days of fever** | 2 | 6 | 2 |
| **Days of hospitalization** | 4 | 9 | 4 |
| **Underlying disease** | NO | NO | NO |
| **Outcome** | Recovery | Recovery | Recovery |

Y=Year.
